# “Hungarian Mine Green”, a Semi‐Natural Copper Pigment from Banská Bystrica Region (Slovakia) ‐ Analytical Evidence and Laboratory Replication

**DOI:** 10.1002/cplu.202500053

**Published:** 2025-05-21

**Authors:** Markéta Žůrková, David Hradil, Janka Hradilová, Petr Bezdička, Silvie Švarcová

**Affiliations:** ^1^ ALMA Laboratory Institute of Inorganic Chemistry of the Czech Academy of Sciences Husinec 1001 Husinec‐Řež 250 68 Czech Republic

**Keywords:** copper pigments, Hungarian mine green, polychrome sculptures, spherulitic malachite, X‐ray powder microdiffraction

## Abstract

A comprehensive approach is taken to investigate the “Hungarian mine green” pigment, including a historical overview, analysis of the pigment on artworks, and its laboratory replication. It is known that in the past, the pigment is collected in wooden reservoirs in which copper (Cu) compounds precipitated from drainage water during copper mining at Špania Dolina–Piesky and Ľubietová deposits, Slovakia. Microsamples of four polychrome wooden sculptures from the 16th‐17th centuries are examined. Posnjakite (Cu_4_SO_4_(OH)_6_·H_2_O) and malachite (Cu_2_(CO_3_)(OH)_2_) are most frequently detected by X‐ray powder microdiffraction. In rock samples from the mine site, brochantite (Cu_4_SO_4_(OH)_6_) and malachite are dominant, and in recent precipitates, only langite (Cu_4_SO_4_(OH)_6_·2H_2_O) is detected, remained in contact with leaking water. In the laboratory, the pigment is prepared by gradually enriching the starting CuSO_4_·5H_2_O solution with NaHCO_3_ in two series of experiments. The initial concentration of the reactants reflected the ratio of Cu^2+^: HCO_3_^−^ = 1:1 found in the mine waters at the site, from which langite crystallizes. However, langite does not formed, while brochantite gradually transformed into posnjakite and subsequently malachite. The co‐occurrence of basic copper sulfates and carbonates and the characteristic grain morphology proved to be the main indicators of the pigment in artworks.

## Graphical abstract

Hungarian mine green, a pigment precipitated from drainage water containing basic copper sulfates and carbonates, represented an important product for painters with the peak of its production in the 16th and 17th centuries in the Banská Bystrica region (Slovakia). Morphological and compositional fingerprints have been described based on microanalysis of Late Gothic artworks and pigment's replication in the laboratory.

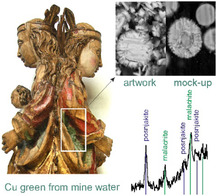

## 1 Introduction

The history of Hungarian mine green is closely related to copper (Cu) extraction in the former Kingdom of Hungary. The most important mining areas were located in the Western Carpathians, specifically in Špania Dolina and L'ubietová in the Banská Bystrica region, Slovakia. Together with Tyrol copper ore mines, the Upper‐Hungarian ones were of the greatest importance in the 14th century in Europe.^[^ ^1^, ^2^ ^]^ Later on, most likely in the second half of the 15th century, production of the Hungarian mine green started as a follow‐up to copper mining. It reached its trading peak in the 16th and 17th centuries, while the production ultimately lasted much longer than the copper mining itself (up to 1950).^[^ ^3^ ^]^

The pigment used to be obtained via precipitation processes from copper sulfate‐rich mine waters leaking from abandoned mine adits. The drainage water was channeled into a cascade of interconnected wooden reservoirs, where the pigment deposition took place. Subsequently, the pigment had been collected once a year.^[^ ^3^, ^4^ ^]^ Such prolonged periods of time were necessary to obtain sufficient pigment yields, as the precipitation and the sedimentation were apparently rather slow due to the relatively low concentration of input ions.

At the beginning of its extraction, the pigment was marketed as “Schifergrün” (the term Schifer probably either refers to German “Kupfer Schiefer”, i.e., copper shale, or to “Schiff”/“schiffen”, i.e., boat to float in reflection to the flowing water from which the pigment precipitated).^[^ ^4^ ^]^ Since the 18th century, the only designation mentioned in historical sources has been “Berggrün” (translated most frequently as “mountain green”). Interestingly, German “Berg” means not only “mountain”, but also (in a figurative sense) “mine”. In this case, it would probably be more fitting to call the pigment a “mine green”. This alternative translation is also used in this paper.

Although the history of this pigment has been described in great detail,^[^ ^1^, ^3^, ^4^, ^5^ ^]^ it has received much less attention from a chemical point of view, especially regarding its composition and characterization. Historical documents show that the pigment used had variable quality with variable composition and, hence, also variable color (from green to blue–green). The color is often ascribed to spherulitic malachite as a dominant mineral in the pigment's composition.^[^ ^2^ ^]^ In order to determine whether spherulitic malachite of the desired deep green color can be formed in the conditions of sulfate mine waters in the Banská Bystrica area, an experiment was carried out in a U‐tube filled with silica gel, where sodium carbonate and copper sulfate solutions diffused against each other. In the middle part of the tube, in the area of supersaturation with Cu^2+^ and CO_3_^2−^ ions, spherulitic malachite precipitated, first small spherules with a rough surface, and then, with a decrease in ionic strength and reaction rate, larger, deep green spherules with a smooth surface.^[^ ^2^ ^]^

However, it can be assumed that natural and slow precipitation from Cu sulfate‐rich mine water under changeable climatic conditions during the years must have resulted in a pigment with more complex composition. This assumption has been pre‐confirmed by analytical results of two samples of the final product extracted around 1800, in which malachite with posnjakite and malachite with brochantite, respectively, were detected.^[^ ^1^ ^]^ In addition, the analyses also showed a small amount of atacamite and paratacamite.^[^ ^1^ ^]^

Such a composition has also been described in paintings. Raman spectroscopy analyses of illuminated manuscripts from Belgium, France, Germany, the Netherlands, and Italy dated to ca. 15th–16th centuries have proved posnjakite and brochantite and malachite in green painted areas.^[^ ^6^ ^]^ The presence of pure brochantite has been revealed by a multi‐analytical study of several Portuguese–Flemish paintings from the 16th century.^[^ ^7^ ^]^ In Czechia, posnjakite was described together with malachite in the 16th century wall paintings, while earlier Gothic murals contain exclusively malachite.^[^ ^8^ ^]^ Previously, the presence of spherulitic malachite has been perceived as a first indicator of artificial production, however, it seems more accurate to focus on the co‐occurrence of basic Cu sulfates and carbonates, as spherulitic malachite often also occurs in nature. Large and irregular malachite spherules have been evidenced, for example, in a wall painting dated to ≈1400 in Lidzbark Warmiński, a castle in northeast Poland.^[^ ^9^ ^]^ In recent years, increasing number of documentation of joint occurrence of basic copper sulfates (such as brochantite, posnjakite, or langite) with spherulitic malachite (Cu_2_(CO_3_)(OH)_2_) in artworks, e.g., in Dutch paintings from the late 15th to 16th century^[^ ^10^ ^]^ has appeared in relation to increasing number of advanced approaches applied in painted artworks’ analysis. It implies that basic copper sulfates were probably more frequent than previously thought.^[^ ^11^, ^12^ ^]^ Although the mixtures of basic Cu sulfates, carbonates, and also Cu chlorides could easily be directly precipitated from stock solutions of various copper salts,^[^ ^8^ ^]^ one has to consider the color that is intended to be obtained. To get green, the old (up to 18th century) recipes mention only *viride salsum* (which can be either copper acetate or also chloride^[^ ^13^ ^]^), neutral verdigris (neutral copper acetate), and green verditer (artificial malachite). None of these recipes starts from Cu sulfates. In the 15th–18th centuries, an artificial malachite was precipitated from Cu(NO_3_)_2_ interacted with CaCO_3_.^[^ ^14^, ^15^, ^16^ ^]^ Therefore, if basic Cu sulfates were not necessary for the artificial preparation of the beautiful green, their presence beside malachite could rather indicate (1) its degradation in the presence of sulfate ions (e.g., from the atmosphere^[^ ^17^, ^18^, ^19^ ^]^) or the natural or semi‐natural origin of pigment obtained, e.g., by mine drainage waters precipitation. Interestingly, there is only one place with historically documented production, the Banská Bystrica area, even though there were many more significant copper deposits in medieval Europe. This may stem from the absence of knowledge, or rather from regionally specific geological and geochemical conditions. Although Tyrol copper green has been sporadically mentioned in the literature, nothing is known about the exact place and technology of its production. It is only mentioned that Hungarian mine green had a more beautiful color in comparison to the Tyrolean one and exceeded it threefold in quality.^[^ ^4^ ^]^

Regarding the drainage waters in the Banská Bystrica area, it is necessary to distinguish two deposits where the green pigment had been produced, which are geologically different. The Špania Dolina–Piesky deposit is situated 1.2 km north of the village of Špania Dolina in the Starohorské Mountains. The Cu(+Ag) ores are dispersed in a hydrothermal stockwork of quartz–siderite–sulfide veinlets and impregnations hosted mainly by Permian sandstones and conglomerates.^[^ ^20^ ^]^ Tetrahedrite is the dominant ore mineral^[^ ^21^ ^]^ with minor amounts of chalcopyrite and other minerals. Gangue minerals include quartz, Fe‐dolomite, siderite, barite, and calcite.^[^ ^20^ ^]^ The Ľubietová–Podlipa Cu–Fe ore deposit is located 5 km east of the village of Ľubietová. The quartz–carbonate (siderite, calcite, ankerite) ore veins are hosted by Paleozoic gneisses and granite porphyry, and the primary Cu minerals are chalcopyrite and tetrahedrite–tennantite.^[^ ^20^ ^]^

Although the host rocks of the two deposits differ, they are poor in carbonates in both cases. However, gangue carbonates may have been the primary source of CO_2_ for acid mine water buffering and malachite precipitation. The geochemistry of drainage waters has not yet been studied in detail, with the exception of one contribution by Majzlan et al.^[^ ^22^ ^]^ focused on the water from Ľubietová. The authors describe it as neutral, with a significant preponderance of Cu^2+^ ions over Fe^2+^ ions. This is probably one of the key factors. Although Cu^2+^ is a common cation of mine waters, as are other cations (such as, e.g., arsenic), iron dominates in most of the world's known deposits where the pH of the water is close to the neutral region. Then, iron oxides adsorb a substantial amount of copper, which prevents the crystallization of pure Cu salts.

And, although acid mine drainage may contain more copper than neutral mine drainage, the higher solubility of minerals in acidic solutions depresses the saturation state even to lower levels. According to Majzlan, the specificity of neutral Cu^2+‐^enriched water should result from such processes occurring at the Ľubietová–Podlipa site that decouple Cu and Fe geochemically in the initial stages of sulfide weathering.^[^ ^22^ ^]^ There is only one other deposit with similar conditions evidenced so far, South Crofty mines (Cornwall, UK, operating from 1592, but no production of Cu pigment is known from here).

The main aim of this work was to simulate formation (precipitation) of the Hungarian mine green, i.e., an expected mixture of basic Cu sulfate(s) and malachite in model system which would reflect the real composition of the mine drainage water in this region as well as to study mineralogical composition of the product(s). In addition, focus was placed on the identification of possible morphological features and designing an approach suitable for the identification as well as characterization of Hungarian mine green in painted artworks.

## 2 Results and Discussion

### 2.1 Microanalysis of Paint Layers

For the purpose of this research, four wooden polychrome sculptures or architectural pieces containing green layers with copper pigments were selected. They were created in the period from the beginning of the 16th to the beginning of the 17th century, when the greatest development of the production of mine green is documented. All of them were produced by workshops in rich mining regions, either a local workshop of the famous artist Master Paul of Levoča (NE Slovakia), or distant workshops active in NW Bohemia on the Czech–Saxon border. The selection of the artworks was based on the high probability of the occurrence of the mine green in connection with the close link between mining and trade among German‐speaking communities in Central Europe. The next selection criterion was to minimize the risk of encountering degradation phenomena that could lead to erroneous interpretations. In polychromy covered with varnish on statues placed indoors (i.e., in a dry atmosphere poor in sulfur), mobilization of sulfate ions due to increased humidity can hardly be expected. On the contrary, wall paintings, where malachite can degrade to sulfates, as already mentioned in the introduction,^[^ ^17^, ^18^, ^19^ ^]^ were not investigated at this stage.

**Figure 1:**
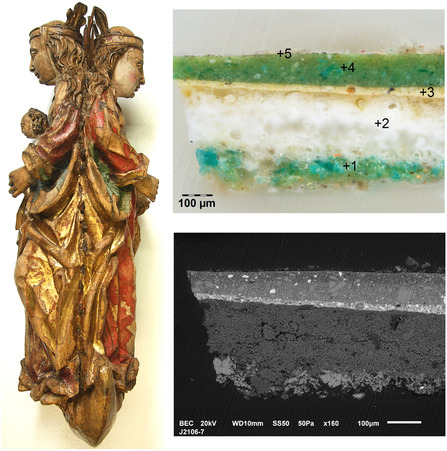
*Candlestick with Virgin Mary and St. Barbara*, around 1500, from the Regional Museum Litoměřice, Czechia (on the left side, photo: O. Trmalová) and cross‐section of the sample 7 from the backside of the Virgin Mary's mantle in visible light (top right, photo J. Hradilová) and back‐scattered electrons (bottom right, photo M. Žůrková); copper green is found in both the original painting (layer 1) and the overpainting (layer 4); a complete description of the layers is given in Table 1.

**Figure 2:**
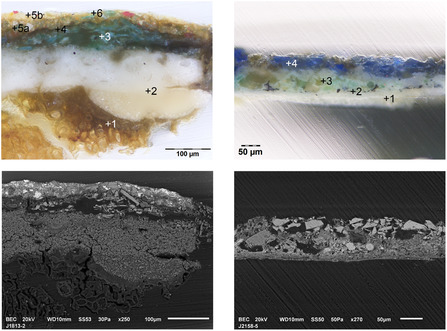
Sample cross‐sections from *Sculptural group of St. George* from Levoča, Slovakia, dated to 1515 and *Renaissance wooden church porch* from Krupka, dated 1609 in visible light (top) and back‐scattered electrons (bottom), showing differences in grain morphology (explanation is given in the text, photo J. Hradilová, K. Šídová, Z. Širillová).

A total of seven microsamples containing green copper pigments were obtained and used for analysis in the form of cross‐sections (embedded in polyester resin), which enable observation and description of the stratigraphy of the layers. In some cases, an untreated fragment was also used for the analysis, but only when the green was the uppermost visible layer available for measurement from the surface. A description of the analyzed artworks and microsamples is given in **Table** **1** together with a simplified description of the layer stratigraphy, which is based on observation in optical microscope (in visible and UV light), measurements by scanning electron microscopy–energy dispersive spectrometry (SEM‐EDS), and Fourier transform infrared (FT‐IR) spectroscopy. Three selected cross‐sections are also shown in **Figure** **1** and **2**.

**Table 1:** Description of painted artworks and studied microsamples containing copper green pigments.

Code	Artwork/Date of creation/ Location/Attribution	Sampling locations	Layering/stratigraphy in the sample cross‐section (from bottom to top), with pigments + binders indicated from SEM‐EDS and μFT‐IR analyses
J2106	*Candlestick with Virgin Mary and St. Barbara* (polychrome wooden carving)/around 1500/Regional Museum Litoměřice, CZ/anonymous master	1 ‐ reverse of the Virgin Mary's mantle, below the hand with the scepter	1. priming – chalk (CaCO_3_)/glue ground 2. tempera painting ‐ copper green pigment with quartz (SiO_2_) and chalk
		7 ‐ back of the Virgin Mary's mantle, under Jesus	1. tempera painting ‐ copper green pigment with admixed quartz, chalk, orpiment (As_2_S_3_), and maybe also minium (Pb_3_O_4_) 2. new priming ‐ chalk/glue ground 3. new underpainting‐ with lead white (2PbCO_3_. Pb(OH)_2_) and chalk delicately tinted with minium and carbon black and tiny grains of copper green pigment (no proteins) 4. overpainting ‐ copper green pigment with admixed lead white and also some earth 5. impurities
		11 ‐ back of the mantle of St. Barbara	1. priming ‐ chalk/glue ground 2. tempera painting ‐ copper green pigment with an admixture of chalk, locally also other pigments, e.g., vermilion (HgS) 3. impurities and retouching
J1813	*Sculptural group of St. George from Levoča* (polychrome wooden sculptures)/around 1515/Parish Church of St. James, Levoča, SK/Master Paul from Levoča	2 ‐ neck of the dragon	1. linden wood with glue insulation 2. priming ‐ chalk/glue ground 3. tempera painting ‐ prevailing copper green pigment 4. surface impurities 5. tempera overpainting ‐ lead‐tin yellow type I (Pb_2_SnO_4_), cinnabar, and copper green pigment admixed 6. varnish
J1725	*St. Mary Magdalene from Stratená* (polychrome wooden sculpture)/1520‐1530/Dedinky filial Church of St. Augustine in Stratená, SK/Master Paul from Levoča and his workshop	6 ‐ back of the sleeve of St. Magdalene's mantle	1. priming ‐ chalk/glue ground 2. proteinaceous insulation 3. tempera painting ‐ copper green pigment, admixture of chalk 4. pigmented varnish with basic copper sulfates and possible admixtures of yellow earths and yellow organic dye
J2158	*Renaissance wooden church porch with Old Testament scenes* (painted wooden architecture)/1609/Church of St. Ann in Krupka, National Heritage Institute at Ústí nad Labem, CZ/Daniel Frank (?)	2 ‐ treetop	1. priming – gypsum (CaSO_4_.2H_2_O) /glue ground 2. tempera painting ‐ gypsum and copper green pigment, admixture of yellow earth 3. impurities
		5 ‐ blue drapery of the stoning soldier	1. priming – gypsum/glue ground 2. charcoal drawing 3. tempera painting ‐ gypsum and chalk colored with copper green pigment 4. tempera painting ‐ smalt (Co‐glass)

The description of the layer stratigraphy (Table 1) shows that the green Cu pigment is found both in the original paint layers and in some overpaintings, which are not precisely dated. Given the gradual replacement of copper green by green earths during the 18th century in the Central European region, it can be roughly estimated that they date back to the 17th century or early 18th century at the latest. Regarding the elemental composition of copper green in the original paintings and overpaintings, it does not differ significantly. Measurements of the green grains by SEM‐EDS show that pure copper carbonate does not occur in any of them, and a small amount of sulfur is always present. If the Cu:S ratio exceeds 10, the grain contains a higher proportion of malachite (there are also cases with Cu:S ratios above 70). If the Cu:S ratio in the grains is averaged, the resulting ratio is close to 4, which corresponds to the Cu:S ratio in the stoichiometric formulas of brochantite or posnjakite (e.g., 3.87 in the sample J1813‐2, 3.96 in J2106‐1, or 4.23 in J2106‐7). Only in the case of sample J2158‐2, the ratio was significantly lower (Cu:S = 2.5), indicating the presence of S also in other compounds, possibly, e.g., calcium sulfate–gypsum. As gypsum is found in the underlying ground layer, it is likely that it has been either (1) mixed into the paint layer as well, or (2) contaminated the paint layer during grinding. It is also worth mentioning that there is a small Cl admixture in the grains, which usually ranges from 0 to 2 at%. The highest Cl contents are in samples J1725‐6, J1813‐2, and J2106‐1, from 2.5 to 4 at%.

In the SEM images in Figure 1 and 2, differences in grain morphology in the green layers are clearly visible. While irregular fragments predominate in both green layers in the sample J2106‐7, elongated laths are clearly visible in sample J1813‐2. In layer 3 of the sample J2158‐5, a small spherule with a significant predominance of Cu (probably malachite) stands out clearly, which contrasts sharply with the irregular fragments of crushed Co glass in the overlying layer.

In order to determine the phase composition of the green layers, six samples were analyzed by X‐ray powder microdiffraction (μXRPD), either in the form of fragments or cross‐sections, as indicated in **Table** **2**. It was confirmed that posnjakite is the most frequent, which corresponds to the most common average ratio of Cu:S = 4 measured in the grains. In one case (J2158‐2), antlerite ‐ Cu_3_(SO_4_)(OH)_4_ was identified next to brochantite, where the stoichiometric ratio of Cu:S is only 3:1, which explains the lower ratio of Cu:S (= 2.5) in the grains of this sample. While the findings of posnjakite and brochantite are not surprising, as they have often been identified in paintings before,^[^ ^2^, ^7^, ^9^, ^10^, ^11^, ^23^ ^]^ antlerite can be considered a rather unique case, besides sporadic occurrences in European painting,^[^ ^23^ ^]^ it is abundantly represented in wall paintings in South America, where it is rather purely natural, available from a local source.^[^ ^24^ ^]^ In addition, the above‐mentioned gypsum is also present in the mixture. It is interesting that the admixture of gypsum was also identified in sample J1813‐2, where gypsum is not used in the ground. It is also possible that gypsum precipitated together with basic Cu sulfates during the production of the green pigment, since Ca^2+^ ions are common in mine waters of the studied area. Furthermore, the increased Cl contents in samples J1813‐2 and J2106‐1 are caused by the presence of atacamite, Cu_2_(OH)_3_Cl, which was identified in both samples. At the same time, it needs to be considered that due to detection limits of μXRPD, atacamite may not be detected in samples with lower Cl contents.^[^ ^25^ ^]^ Speculatively, the increased Cl activity in the paint layer may be due to secondary conservation intervention using chlorine‐containing solvents, although this is not documented in these particular cases.

**Table 2:** Overview of X‐ray powder diffraction (XRPD) and microdiffraction (μXRPD) results and estimation of relative representation of mineral phases.

Sample code/layer	Brochantite Cu_4_SO_4_(OH)_6_	Posnjakite Cu_4_SO_4_(OH)_6_·H_2_O	Langite Cu_4_SO_4_(OH)_6_·2H_2_O	Antlerite Cu_3_(SO_4_)(OH)_4_	Malachite Cu_2_(CO_3_)(OH)_2_	Atacamite Cu_2_(OH)_3_Cl	Others (rock minerals/pigments)
Paint layers							
J1725‐6/3 cross‐section	++	++	–	–	+	+	Q, C
J1813‐2/5 fragment	–	++	–	–	++	+	G, LT, M, Q, C
J2158‐2/2 fragment	+++	–	–	++	–	–	G, Q
J2106‐1/2 fragment	–	+++	–	–	–	–	C, Q
J2106‐7/1 cross‐section	–	+++	–	–	+	–	C
J2106‐7/4 cross‐section	–	++	–	–	++	–	L, Q, C
J2106‐11/2 cross‐section	–	+++	–	–	–	–	C
Rock samples (mining heap Ľubietová)							
HL‐1	++	–	–	++	+	–	D
HL‐2	++	–	–	–	++	–	Q, M
HL‐3	–	–	–	–	+++	–	Q, M, D
HL‐4	++	–	+	–	+	–	Q, M
HL‐5	+	–	–	–	+++	–	Q, M, D
Recent precipitates (Ľubietová)							
RL‐1	–	–	+++	–	–	–	Q, M
RL‐2	–	–	+++	–	–	–	Q, M
RL‐3	–	–	+++	–	–	–	Q, M, F, C, D
Precipitates from experiment 1							
1‐1	+++	–	–	–	–	–	–
1‐2	++	++	–	–	(+)	–	–
1‐3	–	++	–	–	++	–	–
1‐4	–	+	–	–	+++	–	–
1‐5	–	–	–	–	+++	–	–
Precipitates from experiment 2							
2‐1	+++	–	–	–	–	–	–
2‐2	++	+	–	–	++	–	–
2‐3	+	+	–	–	+++	–	–
2‐4	+	(+)	–	–	+++	–	–
2‐5	–	–	–	–	+++	–	–

Note.

+ minor, ++ major, +++ dominant.

Q – quartz SiO_2_, C – calcite CaCO_3_, G – gypsum Ca(SO_4_).2H_2_O, D – dolomite – CaMg(CO_3_)_2_, M – mica group mineral, LT – lead‐tin yellow Pb_2_SnO_4_, L – lead white Pb_3_(CO_3_)_2_(OH)_2_, F – K‐feldspar KAlSi_3_O_8_.

With regard to the possible influence of degradation processes on the composition of the paint layers, their characteristic manifestations were purposefully searched for, knowing that it is not possible to differentiate the primary or secondary origin of sulfates or chlorides from phase analysis alone. No signs of degradation were observed in the paint layers. There were no signs of alteration zonality (depletion or enrichment of certain elements toward the paint surface), no pseudomorphoses (changing, e.g., the Cu:S ratio across the grains), and no signs of microbial activity (e.g., traces of mold). While in the case of atacamite, the secondary origin of chlorine can be at least theoretically accepted with regard to its content in the conservation agents, the secondary origin of sulfur without signs of its migration from the surface (in contact with the atmosphere) or, eventually, from the gypsum‐based ground is rather excluded. Therefore, it can be assumed with a high probability that basic copper sulfates are part of the original pigment.

### 2.2 On‐Site Sampling and Analysis

The composition of the original mine water from which the mine green was precipitated is not known today. From the two locations (Špania Dolina and Ľubietová), only the waters from the Jacobi adit at Ľubietová have been investigated in detail.^[^ ^22^ ^]^ Therefore, the model experiments started with this known composition, and for comparison, rock samples were collected from the Ľubietová–Podlipa deposit, where blue–green copper minerals precipitated on cracks and surfaces, as well as recently formed precipitates, which are still clearly visible on the banks of streams flowing down from the heaps.

**Figure 3:**
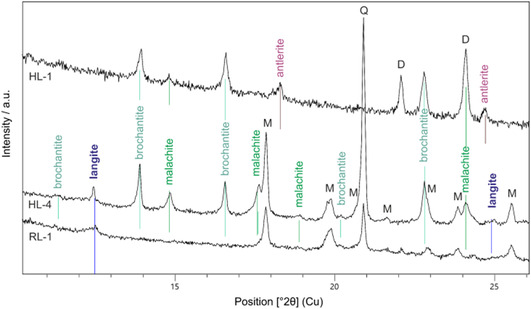
Diffraction patterns representing contemporary precipitates from surface water passing through the heaps at Ľubietová (RL‐1) and green crack fillings of rocks deposited at the same location (HL‐1, HL‐4); M – mica group mineral, Q – quartz SiO_2_, D – dolomite CaMg(CO_3_)_2_.

Unlike Špania Dolina, Ľubietová is known for the recent precipitation of mineral langite, Cu_4_SO_4_(OH)_6_·2H_2_O, e.g., in the above‐mentioned Jacobi adit, which is rather unusual with regard to its thermodynamic instability. According to saturation indices calculated for the Cu_4_SO_4_(OH)_6_ phases in the mine water from the locality, brochantite precipitation was expected.^[^ ^22^ ^]^ However, it is worth mentioning that it was proved experimentally that the blue gel inside the adit converts to langite, i.e., a dihydrated phase of brochantite despite the fact that its calculated saturation index is lower than that of brochantite in this system.^[^ ^22^ ^]^ Langite is considered to be the least stable phase among Cu_4_SO_4_(OH)_6_ minerals, implying that under laboratory conditions, the crystallization of brochantite or posnjakite takes place preferentially, which in turn prevents the formation of langite due to the consumption of Cu and sulfate ions. However, as shown by the results of XRPD measurements performed on collected samples (Table 2, **Figure** **3**), langite is the only newly formed mineral in recent precipitates; the remaining phases (quartz, mica) come from weathered parent rocks concentrated in the sediment. It is interesting that in older dried precipitates in the rock cracks, brochantite with malachite already prevails, and langite was found only in one case. Antlerite ‐ Cu_3_(SO_4_)(OH)_4_ is also occasionally found. Despite the above‐mentioned assumption, it seems likely that langite is formed first, then it gradually turns into brochantite and malachite. Brochantite, malachite, and antlerite were also found in the studied artworks, along with posnjakite, being the most abundant. However, in the rock samples, posnjakite is absent.

### 2.3 Replication of the Pigment – Initial Solution

**Figure 4:**
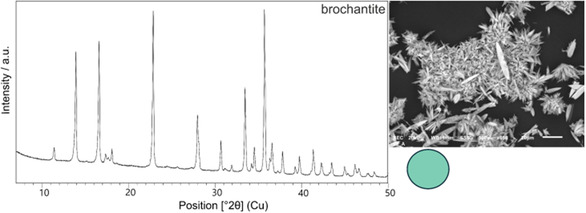
Diffraction pattern of pure brochantite spontaneously precipitated from the initial solution resembling the mine water from Ľubietová; on the right side, crystal morphology by SEM and colorimetric results (*R* = 126, *G* = 206, *B* = 181) are visualized.

Preparation of the green pigment under laboratory conditions was performed in two series (Experiment 1 and 2, respectively) which both started with the initial solution resembling the water from Jacobi adit at Ľubietová, i.e., the concentration ratio of Cu^2+^ and HCO_3_^−^ ions was set to be ≈1:1. The preparation consisted of gradual addition of NaHCO_3_ solution to aqueous solution of CuSO_4_·5H_2_O. Shortly after the first portion of HCO_3_^−^ has been added, the formation of a light bluish gel was observed, which is in good agreement with the in situ observation made by Majzlan et al.^[^ ^22^ ^]^ The turbidity decreased after 24 h, and in ≈ 1 week, it cleared up completely while a light blue–green reaction product sedimented. XRPD analysis of the product proved that brochantite was precipitated (**Figure** **4**), which is in agreement with calculated saturation indices for the analogous system in Lubietová.^[^ ^22^ ^]^ At laboratory temperature, near neutral pH, and in a system simplified in terms of the number of components, no langite was formed. This is consistent with the fact that no successful direct synthesis of langite has been performed to date.^[^ ^26^ ^]^ On the other hand, its indirect synthesis from synthetically prepared rouaite ‐ Cu_4_(OH)_6_(NO_3_)_2_ and by leaching of ktenasite‐type minerals succeeded.^[^ ^27^, ^28^ ^]^ From the above‐mentioned, it seems that there were specific geological and geochemical conditions of the abandoned mine that suppressed the brochantite and/or posnjakite spontaneous crystallization and enabled the formation of langite. Additionally, SEM revealed needle‐shaped crystals with lenticular morphology aggregated into rosettes (Figure 4).

### 2.4 Results of Experiment 1

Considering the low initial concentration of CO_2_ for malachite (Cu_2_CO_3_(OH)_2_) precipitation, the conversion of brochantite to malachite requires the influence of atmospheric CO_2_, which is the driving force of the process. Majzlan et al. assume that CO_2_ required for the conversion is entered from rainfalls in a dissolved form as HCO_3_^−^/CO_3_^2‐^ during the whole year of exposure, which naturally makes the whole open system less defined, especially regarding the resulting composition of the pigment.^[^ ^22^ ^]^

An aqueous solution of NaHCO_3_ was employed as a carbonization agent for experimental purposes, which is described by the following equation.

(1) $${\text{Cu}}_{4}{\text{SO}}_{4}{\left(\text{OH}\right)}_{6}+2{\text{NaHCO}}_{3}\;\to 2{\text{Cu}}_{2}{\text{CO}}_{3}{\left(\text{OH}\right)}_{2}+{\text{ Na}}_{2}{\text{SO}}_{4}+2H_{2}O$$

**Figure 5:**
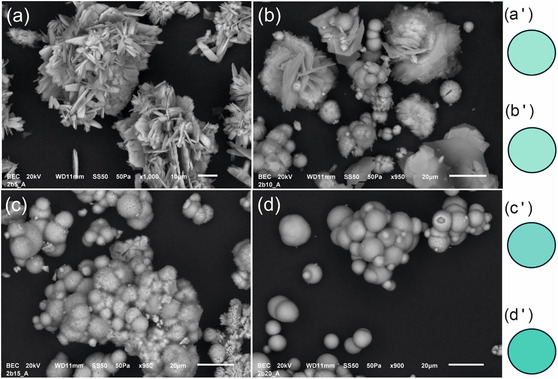
Morphological features of the products of Experiment 1 visualized by SEM from the initial to the last stage: a) brochantite, posnjakite; b) posnjakite, malachite; c) malachite, posnjakite; d) malachite, and visualization of the colorimetric results obtained in *RGB* values for corresponding products. a') *R* = 138, *G* = 201, *B* = 186; b') *R* = 138, *G *= 201, *B* = 186; c') *R* = 100, *G* = 186, *B* = 176 and d') *R* = 43, *G* = 177, *B* = 158; photo M. Žůrková.

The XRPD results of Experiment 1 products have revealed that the conversion from brochantite to malachite takes place through posnjakite (Cu_4_SO_4_(OH)_6_·H_2_O) formation (Table 2). It should be mentioned that upon detailed examination of the diffraction pattern of the first sample, a subtle indication of malachite is also observable in this case, but its amount is at the limit of detection. The morphological features of the products differ significantly from each other, as shown in the SEM images depicted in **Figure** **5**.

In contrast to lenticular‐shaped crystals of brochantite, posnjakite is characterized by lath‐like crystals in rosette arrangement. While in the initial stage of the conversion, it is accompanied by brochantite, in the latter stage by spherulitic crystal aggregates of precipitated malachite. In the last sample with the highest concentration of HCO_3_^−^, only spherules of malachite were observed. Colorimetric parameters in RGB show a gradual darkening of the blue–green color of the pigment (Figure 5).

### 2.5 Results of Experiment 2

**Figure 6:**
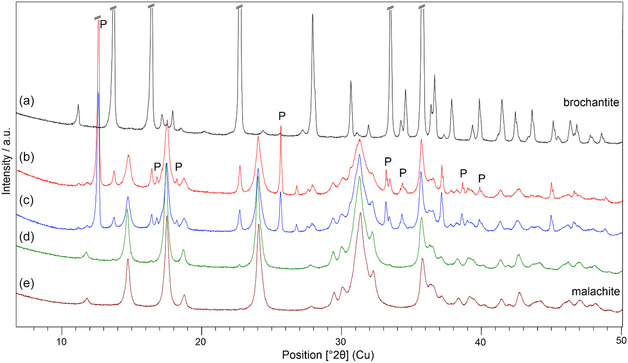
Diffraction patterns of the model pigments prepared in the Experiment 2: a) sample 2‐1 representing the initial solution; solely brochantite precipitated, b,c) samples 2‐2 and 2‐3, respectively; posnjakite (P) detected together with brochantite and malachite, d) sample 2‐4 with prevaining malachite, and e) sample 2‐5; solely malachite detected; the overview of XRPD results are listed in Table 2.

The goal of Experiment 2 was to perform the whole conversion of brochantite → malachite in one system, which would mimic the process more authentically with respect to the formation of the green pigment. In this experiment, the changes in mineralogical composition and grain morphology were continuously monitored (v Experimental). The XRPD results are presented in **Figure** **6**. In comparison to the isolated systems (one‐time additions of NaHCO_3_ Experiment 1), in this arrangement, a small amount of malachite was clearly detected already after the first addition of HCO_3_^−^ to the initial solution.

Furthermore, brochantite phase remained in the product for almost the whole experiment (detected in samples 1–4), and posnjakite was present only in samples 2 and 3 in a relatively smaller amount than in the case of Experiment 1. These differences can be caused by gradual conversion within one system in which brochantite was crystallized and stabilized first. The persisting presence of basic Cu sulfates indicates that they should be expected in some amount even in malachite‐rich pigments prepared by precipitation from sulfate‐containing waters. Products of the second experiment contained bigger crystals, which is probably due to a larger amount of the reactants used in order to obtain higher reaction yield (the concentration ratio was maintained). The crystal size is probably responsible for the more intense greenish color of the products in comparison to Experiment 1. Nevertheless, it is obvious that the color of the model pigment is rather light and pale in comparison to the description of Hungarian mine green pigment reported in various historical documents.^[^ ^1^, ^3^, ^4^, ^5^ ^]^ This pigment was valued for its color, which has often been likened to malachite, i.e., intense green. This may be related to the prolonged time of the continuous inflow of mine water before pigment collection (≈1 year).^[^ ^5^ ^]^ It can be supposed that a much higher amount of the pigment collected, as well as particle size in the real system, contributed to its color intensity in comparison to the laboratory preparation.

### 2.6 Brochantite vs. Posnjakite

As it follows from the experiments, the system is not very robust even under laboratory conditions. For instance, the addition of HCO_3_^−^ dropwise versus direct pouring of solutions affects the reaction time of the conversion (i.e., the time that elapses until the solution above the sediment clears, where no turbidity is observed anymore). In the case of dropwise additions, the turbidity cleared up earlier than in the case of direct pouring (in days). Moreover, some of the samples took longer to sediment completely, even though their composition and preparation procedure were identical. These factors have a direct effect on the representation of individual mineralogical components within its composition and an indirect effect on morphology as well as the color of the product. The above‐mentioned observations are connected especially with the representation of posnjakite. Zittlau et al. have performed a series of experiments to investigate the stability of brochantite and posnjakite (as well as other basic copper sulfates) based on acid‐solution calorimetry determination of the formation enthalpies.^[^ ^26^ ^]^ They have found out that posnjakite has no stability field in the system CuO–SO_3_–H_2_O, therefore, it is not possible to set the range of specific conditions over which it would be stable, but it is always metastable with respect to brochantite. Their data‐based evaluation of Gibbs free energies of posnjakite with respect to brochantite corresponds to a frequent occurrence of posnjakite in brochantite syntheses.^[^ ^26^, ^29^, ^30^, ^31^ ^]^ Furthermore, posnjakite transforms into brochantite by ageing if it is left in contact with the aqueous solution. It is in agreement with here presented experiments and also with the composition of crack fillings in rock samples, where posnjakite is missing. However, posnjakite may have been common in commercially available mine green pigment, as evidenced by analyses of artworks.

The variable concentrations of CO_2_ in the atmosphere, the frequency of precipitation during the year, the concentration of Cu^2+^ in the leaking water, etc., are other factors influencing the mineralogical composition of the resulting pigment, its grain size, and color. Therefore, the qualitative properties of this pigment could not be constant. It has been reported that the pigment was sold in 3 grades of quality. The first was a low‐grade pigment found in the reservoirs at the upper parts of the cascade (coarse and often mixed with sand), the second grade was finer and deposited in lower reservoirs, and the third grade was the finest one, found in reservoirs at the end of the cascade.^[^ ^1^ ^]^

### 2.7 How to Recognize Mine Green in a Painting

As shown by experiments, the gradual increase in dissolved CO_2_ (supplied to the reservoir, for example, in the form of rainfall) leads to gradual conversion of brochantite (which is the only phase crystallizing directly from untreated mining water) through posnjakite to malachite. Therefore, the resulting pigment is usually a mixture of these phases in various proportions, which exactly corresponds to the analyses of samples taken from works of art. It should be noted that another basic copper sulfate, antlerite, was also found in copper greens in paints. However, it occurs only rarely and was found analogously in the fillings of cracks after flowing water in rocks at the mining site. More interesting is the systematically appearing and variable content of chlorine in the painting layers, related to the presence of atacamite. For example, in wall paintings, atacamite can typically be a product of degradation, where chlorine is brought in by rising moisture.^[^ ^8^ ^]^ However, on polychrome sculptures studied here, no such conditions and signs of degradation are evident, and the origin of chlorine is thus unclear. One possible explanation for its presence on polychrome sculptures could be secondary conservation interventions, when organic solvents containing chlorine were used for cleaning and removing overpaintings. Alternatively, its origin in the mine green itself cannot be ruled out, since the only remains from historical pigment production analyzed so far contain atacamite as an admixture.^[^ ^1^ ^]^

**Figure 7:**
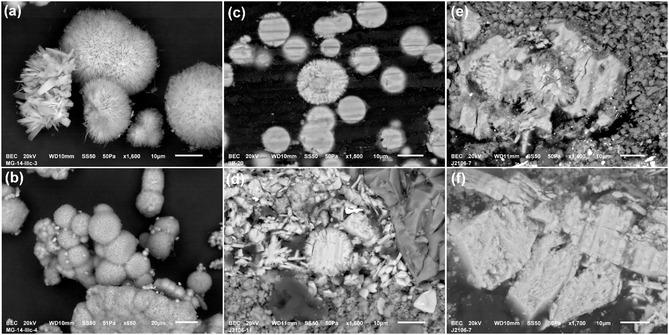
A detailed morphological features of Cu green pigment captured in the model samples (a–c) and in microsamples from *Candlestick with Virgin Mary and St. Barbara* (d–f); aggregates of spherulitic malachite particles together with rosette arrangement of lath‐like crystals of basic Cu sulfates appear both in the model (a) and similarly in artwork (d), and the same sun/flower appearance of malachite particles are recognized in the cross‐sections of model precipitate (c) and paint layer (d); in artworks, however, the spherules can be crushed (e) and the laths merged together to irregular grains as a result of drying and solidification (f); photo M. Žůrková.

Perhaps the most important clue for recognizing mining green when studying microsamples is the morphological features of the grains visible at high magnification. Sulfates crystallizing from water usually have a lath‐like or lenticular shape and form rosette‐like aggregates. However, the key feature is the frequent presence of tiny malachite spherules (only 10–20 μm large), which are actually needlelike aggregates growing around a crystallization nucleus. In cross‐section, they resemble a sun (**Figure** **7**). As can be seen from the picture, completely identical spherules were found in the prepared pigment and in the microsamples of paints. Unfortunately, it often happens that the spherules are broken due to rubbing/grinding of the pigment, and irregular aggregates are also a frequent phenomenon, hardened pieces of dried pigment, where the morphology of the individual grains is no longer visible.

## 3 Conclusions

The copper pigment formed by precipitation from mine waters (“Hungarian mine green”) contains basic copper sulfates (most often posnjakite and/or brochantite) and copper carbonates (malachite). These phases were found both in the studied artworks and in precipitates from mock‐up solutions replicating the composition of the mine water in Ľubietová, Slovakia, gradually enriched with HCO_3_^−^, which in the real conditions of historical pigment production entered the system, e.g., in rainwater. Another similarity between the experiments and the analysis of artworks is the morphology of the pigment grains in both cases, it is possible to recognize lath‐shaped or lenticular crystals of basic copper sulfates, sometimes forming rosettes, as well as needle‐shaped aggregates of malachite in the form of small spherules (≈10–20 μm in diameter), which resemble sunflower in the cross‐section. However, recognizing the pigment in a painting by morphology can be difficult if the spherules are disintegrated after the pigment has been treated by rubbing, or form compact clumps because the pigment has been dried and solidified.

While in the experiments, posnjakite is a transient, unstable phase (when HCO_3_^−^ concentration is increased, brochantite transforms through posnjakite to malachite), in the studied polychrome sculptures, posnjakite was the most frequently represented basic Cu sulfate. However, in general, even relatively pure malachite has a small amount of sulfur remaining at the end of the process, and therefore, the co‐occurrence of Cu carbonates and basic Cu sulfates is another distinguishing feature of this pigment. To be sure of the identification, the complementary use of μXRPD for phase microanalysis of paint layers and electron microscopy to describe grain morphology in high resolution is highly recommended.

Other phases can rarely appear among basic Cu sulfates, such as langite or antlerite, which were described in rock samples and recent precipitates directly at the mining site. Antlerite was found in one of the selected artworks studied here, but langite was not. In all studied artworks, copper green has identical characteristics, and it is therefore likely that it is the same pigment, albeit with variable composition. Therefore, it seems to be clear that in the 16th and early 17th centuries the “Hungarian mine green” was available in Saxon communities operating both in the NE Slovakia (Spiš region) and W Bohemia, on the Czech–Saxon border. μXRPD has proven to be a robust method for unambiguously distinguishing between compositionally similar basic copper sulfates, and its use will be essential in further comparative research of copper salts of any origin in paints. Comparative studies using μXRPD will also need to be carried out in the future to determine the extent of the use of the Hungarian mine green in paints based on the here described materials’ signatures.

## 4 Experimental Section

4.1

4.1.1

##### Model Experiments: Preparation of the Initial Solution

0.67 g of CuSO_4_ · 5H_2_O was dissolved in 100 mL of distilled water (yielding a concentration of 2.68 · 10^−2^ M). 1.1 g of NaHCO_3_ was dissolved in 50 mL of distilled water (yielding a concentration of 2.6 · 10^−1^ M), representing a stock solution. 10 mL of NaHCO_3_ was gradually drop‐added to the solution of CuSO_4_ · 5H_2_O. The concentration ratio of c_(Cu2+)_:c_(HCO3−)_ was ≈1:1. This initial solution was stirred thoroughly and left under laboratory conditions. The pH of the initial solution was 6.8. For comparison, the pH of the reference water sample from Lubietová analyzed by Majzlan et al was 6.43.^[^ ^22^ ^]^ In the next 24 h, the light blue turbidity cleared up, and the light green–blue product started to sediment. The enrichment of the initial solution by CO_2_ consisted of further addition of NaHCO_3_ at different concentrations, which was performed in two different ways to simulate the gradually increasing influence of CO_2_ on the water retained in outdoor reservoirs. CuSO_4_ · 5H_2_O and NaHCO_3_ were both of p.a. quality and purchased from Lachner.

##### Model Experiments: Experiment 1

In the first series of the experiment (Experiment 1), five initial solutions were prepared. A different amount of NaHCO_3_ (5, 10, 15, and 20 mL) were added into each model system yielding its concentration in the first sample 3.4 · 10^−2^ M, 4.3 · 10^−2^ M in the second one, 5.2 · 10^−2^ M in the third one and 6 · 10^−2^ M in the fourth one, respectively. The resulting mixture was stirred thoroughly and left under laboratory conditions for one week until the solution above the sediment cleared up. After that, the settled product was washed in distilled water and spun in a laboratory centrifuge. The whole procedure was done three times, then the samples were allowed to evaporate under laboratory conditions and subsequently used for analysis (XRPD, SEM‐EDS).

##### Model Experiments: Experiment 2

In the second series of the experiment (Experiment 2), the reaction was performed in one system, i.e., simulating the natural process more precisely, in contrast to the isolated model systems in the first series. Five initial solutions (model systems) were prepared, and NaHCO_3_ was gradually poured in a parallel arrangement according to **Table** **3**. To increase the amount of the final product, the amount of the reactants was multiplied by a factor of 5 in comparison to Experiment 1, i.e., 3.35 g of CuSO_4_ · 5H_2_O was dissolved in 500 mL of distilled water. The stock solution of NaHCO_3_ was prepared by dissolving 5.5 g of NaHCO_3_ in 250 mL of distilled water. 50 mL of NaHCO_3_ was added to the solution of CuSO_4_ · 5H_2_O. The concentrations of the reactants were the same as in Experiment 1. The additions of NaHCO_3_ stock solution were the same throughout this experiment: 25 mL. The time between additions was 1 week. After that time, a control sample was taken and investigated by means of XRPD and SEM‐EDS to monitor the composition and morphological features. The products were washed in distilled water, centrifuged, and allowed to evaporate before the analysis. All experiments were processed under laboratory temperature (20 °C).

**Table 3:** Arrangement of the additions of NaHCO_3_ (25mL, 2.6 · 10^−1^ M) in the Experiment 2. The time between additions was 1 week.

Model system	Addition 1	Addition 2	Addition 3	Addition 4
1	–	–	–	–
2	+	–	–	–
3	+	+	–	–
4	+	+	+	–
5	+	+	+	+

##### Light Microscopy (LM)

Microsamples of the artworks were embedded in polyester resin and ground into cross‐sections. Observations were carried out by Olympus BX‐60 light microscope in reflected visible and UVA light with a 330–380 nm wavelength range. Layer stratigraphy was described based on the material color and luminescence in UV mode.

##### X‐Ray Powder Diffraction (XRPD): Conventional Bragg–Brentano X‐Ray Powder Diffraction Measurements

Diffraction patterns were collected with a PANalytical X´Pert PRO diffractometer equipped with a conventional X‐ray tube (CuKα radiation, 1. 5409 Å, 40 kV, 30 mA, line focus) and a linear position‐sensitive detector PIXCel with an anti‐scatter shield. X‐ray patterns were measured in the range of 7°–90° 2Θ with a step of 0.0131° and 200 s counting per step. Conventional Bragg–Brentano geometry was used with the following parameters: 0.04 rad Soller slit, 0.5° divergence slit, and 15 mm mask in the incident beam, 0.5° anti‐scatter slit, 0.04 rad Soller slit and Ni beta filter in the diffracted beam. Silicon zero background sample holders were used to accommodate small amounts of precipitates. The duration of the scan: ≈1 h 28 min.

##### X‐Ray Powder Diffraction (XRPD): X‐Ray Powder Microdiffraction Measurements

Diffraction patterns were collected with a PANalytical X´PertPRO MPD diffractometer equipped with a conventional X‐ray tube (CoKα radiation, 1.7890 Å, 40 kV, 30 mA, point focus). A glass collimating mono capillary with a length of 165 mm and an exit diameter of 0.1 mm was used in the primary beam. A multichannel position‐sensitive detector, X’Celerator, with an anti‐scatter shield and the Fe beta filter, was used in the diffracted beam. X‐ray patterns were taken between 4° and 80° 2Θ with a 0.0334° step and 2200 s counting time per step, which produces a total counting time of about 12 h. Patterns were not pretreated before interpretation.^[^ ^25^, ^32^ ^]^

##### X‐Ray Powder Diffraction (XRPD): Evaluation of X‐Ray Patterns

Qualitative analysis was performed with the HighScorePlus software package (Malvern PANalytical, The Netherlands, version 5.2.0) together with the PDF‐5+ database.^[^ ^33^ ^]^ The line profile analysis was performed using routines implemented in the HighScorePlus software.^[^ ^34^ ^]^

##### Scanning Electron Microscopy with Energy‐Dispersive Spectrometry (SEM‐EDS)

Morphological features of selected cross‐sections and model samples were investigated using the scanning electron microscope Jeol JSM6510 equipped with the energy‐dispersive spectrometer INCA (Oxford Instruments) with an SDD detector allowing detection of elements heavier than Be at 125 eV resolution. Using this SEM, measurements were carried out in low vacuum mode under 50 Pa pressure, and back‐scattered electrons were detected. The applied accelerating voltage was 20 kV.

##### Colorimetry

A portable colorimeter, Ninja TB 01 (Ninja Color), based on measurement of reflected radiation, was used for colorimetric characterization of the experimentally prepared samples. A sufficient amount of the sample was finely smoothed onto a microscopic glass placed onto a white mat, overlaid by PEEK film, and measured immediately. Measuring range of the colorimeter is: *R* = 0–255, *G* = 0–255, *B* = 0–255, with the accuracy of ≈1.5% in each *RGB* channel. The RGB color filters are complemented by an IR filter that crops with 90–95% efficiency wavelengths >650 nm.

##### Fourier Transform Infrared (μ)Spectrometry (μFTIR) and Staining Tests

In order to identify the organic binders in the paint layers, the cross‐sections were analyzed by attenuated total reflection technique (FTIR‐ATR) on an FTIR microscope (Hyperion 3000 with a VERTEX 70 spectrometer; Bruker, Germany). Number of scans per spectrum equaled 64, with a resolution of 4 cm^−1^; the measured spectral region range was 4000–500 cm^−1^. Interpretation was performed using Opus 8.0 software. As a complementary method, staining tests with Lugol S were used to map the distribution of proteins.

## Conflict of Interest

The authors declare no conflict of interest.

## Data Availability

The data that support the findings of this study are available from the corresponding author upon reasonable request.
